# Dual energy CT findings in gout with rapid kilovoltage-switching source with gemstone scintillator detector

**DOI:** 10.1186/s41927-019-0104-5

**Published:** 2020-01-17

**Authors:** Elin Svensson, Ylva Aurell, Lennart T. H. Jacobsson, Anton Landgren, Valgerdur Sigurdardottir, Mats Dehlin

**Affiliations:** 1 Department of Radiology at Institute of Clinical Sciences, Sahlgrenska Academy, Gothenburg, Sweden; 2 Department of Rheumatology and Inflammation Research, Sahlgrenska Academy, Gothenburg, Sweden; 3 Center of Clinical Research (CKF) Dalarna, Uppsala University, Uppsala, Sweden

**Keywords:** Gout, Dual energy CT, Monosodium urate crystals, Foot, Diagnostic imaging

## Abstract

**Background:**

A definite diagnosis of gout requires demonstration of monosodium urate crystals in synovial fluid or in tophi, which in clinical practice today seldom is done. Dual energy CT (DECT) has repeatedly been shown to be able to detect monosodium urate crystals in tissues, hence being an alternative method to synovial fluid microscopy. The vast majority of these studies were performed with CT scanners with two X-ray tubes. In the present study we aim to investigate if and at what locations DECT with rapid kilovoltage-switching source with gemstone scintillator detector (GSI) can identify MSU crystals in patients with clinically diagnosed gout. We also performed a reliability study between two independent readings.

**Methods:**

Patients with new or established gout who had been examined with DECT GSI scanning of the feet at Sahlgrenska University Hospital, Mölndal between 2015 and 2018 were identified. Their medical records were sought for gout disease characteristics using a structured protocol. Urate deposits in MTP1, MTP 2–5, ankle/midfoot joints and tendons were scored semiquantatively in both feet and presence of artifacts in nail and skin as well as beam hardening and noise were recorded. Two radiologists performed two combined readings and scoring of the images, thus consensus was reached over the scoring at each occasion (Espeland et al., BMC Med Imaging. 2013;13:4). The two readings were compared with kappa statistics.

**Results:**

DECT GSI could identify urate deposits in the feet of all 55 participants with gout. Deposits were identified in the MTP-joints of all subjects but were also present in ankle/midfoot joints and tendons in 96 and 75% respectively. Deposition of urate was predicted by longer disease duration (Spearman’s Rho 0.64, *p* < .0001) and presence of tophi (*p* = 0.0005). Artifacts were common and mostly found in the nails (73%), a minority displayed skin artifacts (31%) while beam hardening and noise was rare. The agreement between the two readings was good (Κ = 0.66, 95% CI = 0.61–0.71).

**Conclusion:**

The validity of DECT GSI in gout is supported by the identification of urate in all patients with clinical gout and the good correlations with clinical characteristics. The occurrence of artifacts was relatively low with expected locations.

## Introduction

Gout is world wide the most common inflammatory arthritis and is caused by the deposition of monosodium urate (MSU) crystals in joints, tendons and various tissues which may cause an inflammatory response, clinically recognized as an acute episode of gout. The gold standard for diagnosis is the demonstration of MSU crystals in the synovial fluid of an affected joint or in larger deposits of urate, so called tophi. However, in clinical practice joint aspiration is seldom performed [1] and the majority of patients do not have visible tophi. This is reflected in the ACR/EULAR classification criteria for gout from 2015 where imaging evidence of urate deposition by ultrasound or dual energy CT (DECT) is included as an important item [2].

DECT has been shown capable of detecting MSU crystals with high precision in many studies [3–6].

However, the vast majority of these studies were performed on CT scanners with two X-ray tubes (dual source) while the performance of other technical CT solutions are much less studied. As of today, there are five different types of DECT scanners available: dual source DECT, twin-beam single-source CT with gold filter, rapid kilovoltage-switching source with gemstone scintillator detector (GSI), dual-layer multidetector DECT, and dual-scan single source [7, 8].

Of course, detection of urate deposits with DECT does not come without artifacts, which potentially could lead to misclassification. Not all material color coded as urate on DECT images corresponds to likely or actual deposits of urate. Reported sites of such artifacts include the skin, nose, calluses and nail bed [7]. Other recorded artifacts that might result in false coloring at the DECT image are motion artifacts, noise and beam hardening artifacts [7]. Noise is a randomly unwanted change in pixel values which in the DECT context means that a single pixel being colored might be considered false. The signal to noise ratio (S/N) varies significantly between different technical solutions throughout the imaging process and is always of concern while assessing radiological image quality. Beam hardening is an artifact due to the phenomenon that low energy x-ray photons do not always pass the whole way through the patient and thus the x-ray beam becomes “hardened” i.e. more energetic during its passage, which in particular is seen in relation to metallic implants and sometimes in relation to bone cortex.

There are great differences between the different technical DECT solutions. In the present study, all examinations were performed with a single source system (DECT GSI) in which the tube switches between 80 kVp and 140 kVp within less than 0.03 μs. The special scintillator detector (Gemstone®) can read the data separately with very low afterglow, which gives a very high spatiotemporal resolution. The dual source technique requires much longer time to obtain the two data sets (about70 ms) and they are initially reconstructed separately by filtered back projection and the material decomposition is performed afterwards. With the DECT GSI technique the time between the two energy projection levels is so short that it enables another algorithm for material decomposition, which is done directly and using raw data. Theoretically this technical solution will give not only a more accurate material decomposition information but should be more robust for artifacts such as beam-hardening and motion artifacts [8].

In the present study we wanted to investigate if DECT GSI could detect MSU crystals in patients with clinically diagnosed gout and to correlate the findings against clinical characteristics of gout. We also performed a reliability study between the two combined readings of DECT GSI images.

## Methods

Patients with new or established gout who all fulfilled the ACR/EULAR classification criteria for gout [2] and had been examined with DECT GSI scanning of the feet at Sahlgrenska University Hospital, Mölndal between 2015 and 2018 as part of clinical diagnostics or disease monitoring of gout were identified. They all had at least one ICD-10 diagnosis of gout (M10) in their medical records. Their medical records were examined according to a structured protocol for gout disease characteristics including information on onset of disease, presence of tophi, comorbidities (ischemic heart disease (IHD), hypertension (HT), dyslipidemia), treatment with loop or thiazide diuretics or urate lowering therapy (ULT), body mass index (BMI), serum urate level at time of DECT, and renal function described as eGFR calculated by the CKD-EPI formula [9].

All radiographic examinations were performed using a single source dual-energy CT system (Discovery CT750HD, GE Healthcare, Milwaukee, WI, USA) with 64 detectors and a scan field of view of 32 cm. The tube voltage of the system is switched extremely rapidly, in less than 0.03 μs, between the two different energy levels i.e. 80 kVp and 140 kVp, both at 550 mA. The slice thickness was 0.625 mm, pitch 0.516 and rotation time 0.8 s.

All patients were scanned with the feet resting at the gantry-table, holding the knee in a flexed position and the scanning volume included the whole foot and ankle bilaterally.

Urate deposits in MTP1, MTP 2–5, ankle/midfoot joints and tendons were scored semiquantatively in both feet in the following manner: 0 = no deposit, 1 = dots, 2 = single deposit and 3 = more than 1 deposit, thus a maximum score of 12 per foot [10]. Presence of artifacts in nail and skin as well as beam hardening and noise were also identified and recorded. All images were examined by one experienced radiologist and one junior radiologist who read and scored all images together. If disagreement, images were read together until consensus was reached [11]. This procedure was performed twice.

DECT GSI findings for the total score of urate depositions in the joints and tendons in both feet were analyzed according to demographics (age and gender), features of gout (disease duration, presence of tophi, presence of joint erosions, urate levels at time of DECT defined as low ≤405 μmol/L and high > 405 μmol/L), diuretic and ULT use, BMI defined as high ≥30 and renal function by eGFR in the following categories: > 90, 60–90 and < 60 ml/min per 1.73m^2^.

Baseline characteristics are expressed as absolute counts and proportions for categorical variables, and as means ± standard deviations for continuous variables. The Wilcoxon Mann-Whitney test and Chi-2 tests were used when appropriate to study associations between subgroups and study variables (total urate deposits, age, gender, presence of tophus, erosive disease, disease duration, urate level at DECT, loop or thiazide diuretic use, BMI, renal function). Spearman correlation coefficients were used to assess correlations between total urate deposits on DECT GSI and continuous factors (disease duration, serum urate). BMI was defined as high if ≥30 kg/m^2^, which corresponds to the internationally accepted definition of obesity. Correlation coefficients above the threshold of 0.30 were considered relevant and nominal *p*-values < 0.05 were considered statistically significant. Agreement between the two readings of DECT images were evaluated by kappa statistics.

## Results

We identified a total of 55 patients with a clinical diagnosis of new or established gout who had been examined with DECT GSI scanning of both feet, 43 men and 12 women, with a mean age of 60 (SD = 16) years for the men and 53 (SD = 14) years for the women (Table 1). All patients fulfilled the ACR/EULAR classification criteria for gout [2]. Mean disease duration was 7 years (SD = 7), presence of tophi was more common among the women while erosive disease was more common in the men (Table 1). Comorbidities such as HT, IHD and dyslipidemia were more common in men while obesity was much more common among the women, 75% compared to 35% of the men (Table 1). A minority of the patients, 40%, were on ULT (allopurinol) at the time of DECT GSI examination with a mean dose of 210 mg daily. The total population had a mean serum urate level above the recommended target level, 360 μmol/L, irrespective of whether they were on ULT (40%) or not. (Table 1). A third of the patients had eGFR below 60 mL/min/1.73m^2^ (Table 1).

**Table 1:** Characteristics of study population overall and by gender

	Total, n = 55	Men, n = 43	Women, n = 12
Age, mean years (SD)	58 (15)	60 (16)	53 (14)
Disease duration, mean years (SD)	7 (7)	6 (7)	9 (8)
Tophus, n (%)	7 (13)	4 (9)	3 (25)
Erosive disease, n (%)	36 (65)	29 (67)	7 (58)
IHD, n (%)	15 (29) *n* = 51	12 (29), *n* = 42	3 (33), *n* = 9
HT, n (%)	29 (54) *n* = 54	23 (53), *n* = 43	6 (55), *n* = 11
Dyslipidemia, n (%)	15 (29) n = 51	14 (33), *n* = 43	1 (11), *n* = 9
Diuretic use, n (%)	16 (29)	12 (28)	4 (33)
ULT at DECT, n (%)	22 (40)	16 (37)	6 (50)
BMI high ≥30, n (%)	20 (48) n = 42	12 (38), *n* = 32	8 (75), *n* = 10
Urate at DECT, μmol/L, mean (SD)	457 (134)	465 (133)	424 (137)
Urate at DECT, μmol/L, mean (SD) *with* ULT, *n* = 22	405 (114)	401 (93), *n* = 16	418 (168), *n* = 6
Urate at DECT, μmol/L, mean (SD) *without* ULT, *n* = 33	491 (137)	504 (140), *n* = 27	429 (113), *n* = 6
eGFR > 90, n (%)	16 (29)	12 (28)	4 (33)
eGFR 60–90, n (%)	20 (36)	15 (35)	5 (42)
eGFR < 60, n (%)	19 (35)	16 (37)	3 (25)

Abbrevaition: SD = standard deviation, IHD = ischemic heart disease, HT = hypertension, ULT = urate lowering treatment, BMI = body mass index

**Fig. 1:**
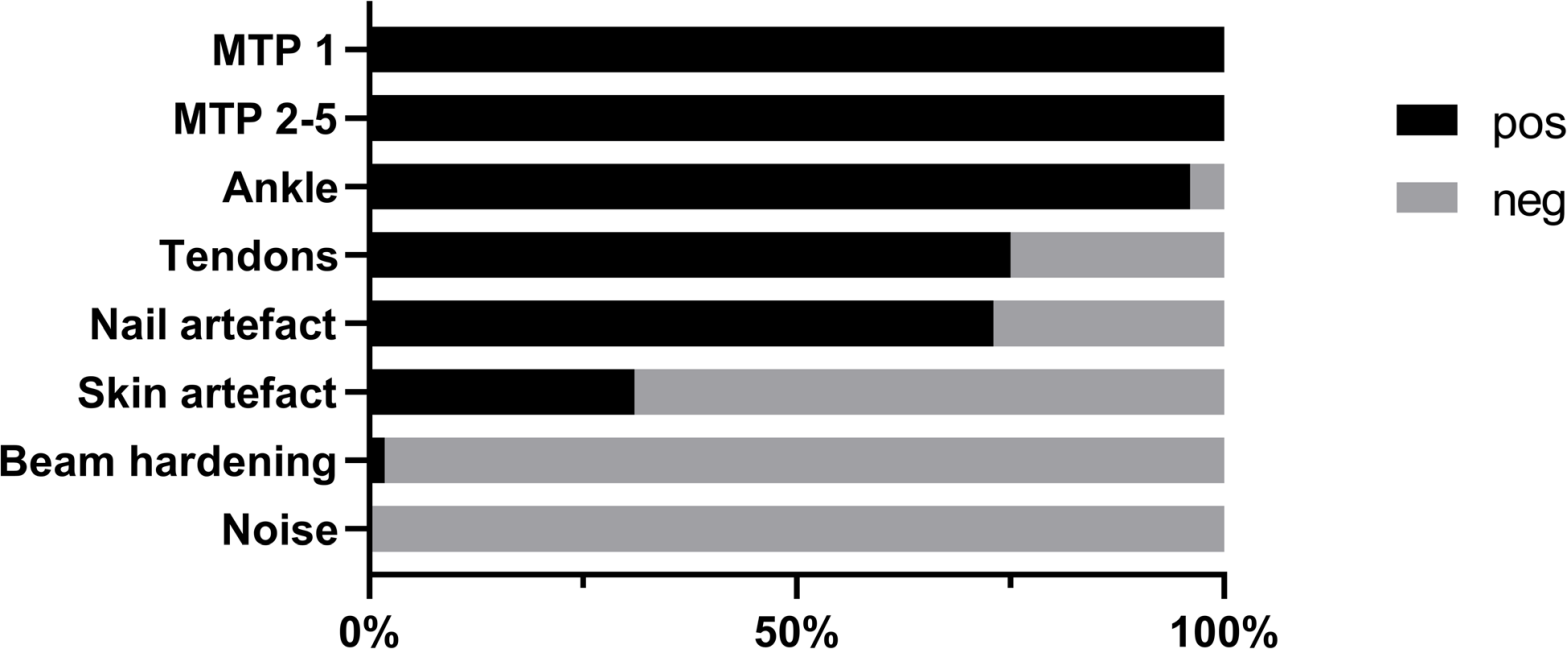
Occurrence of urate deposits and artifacts

**Fig. 2:**
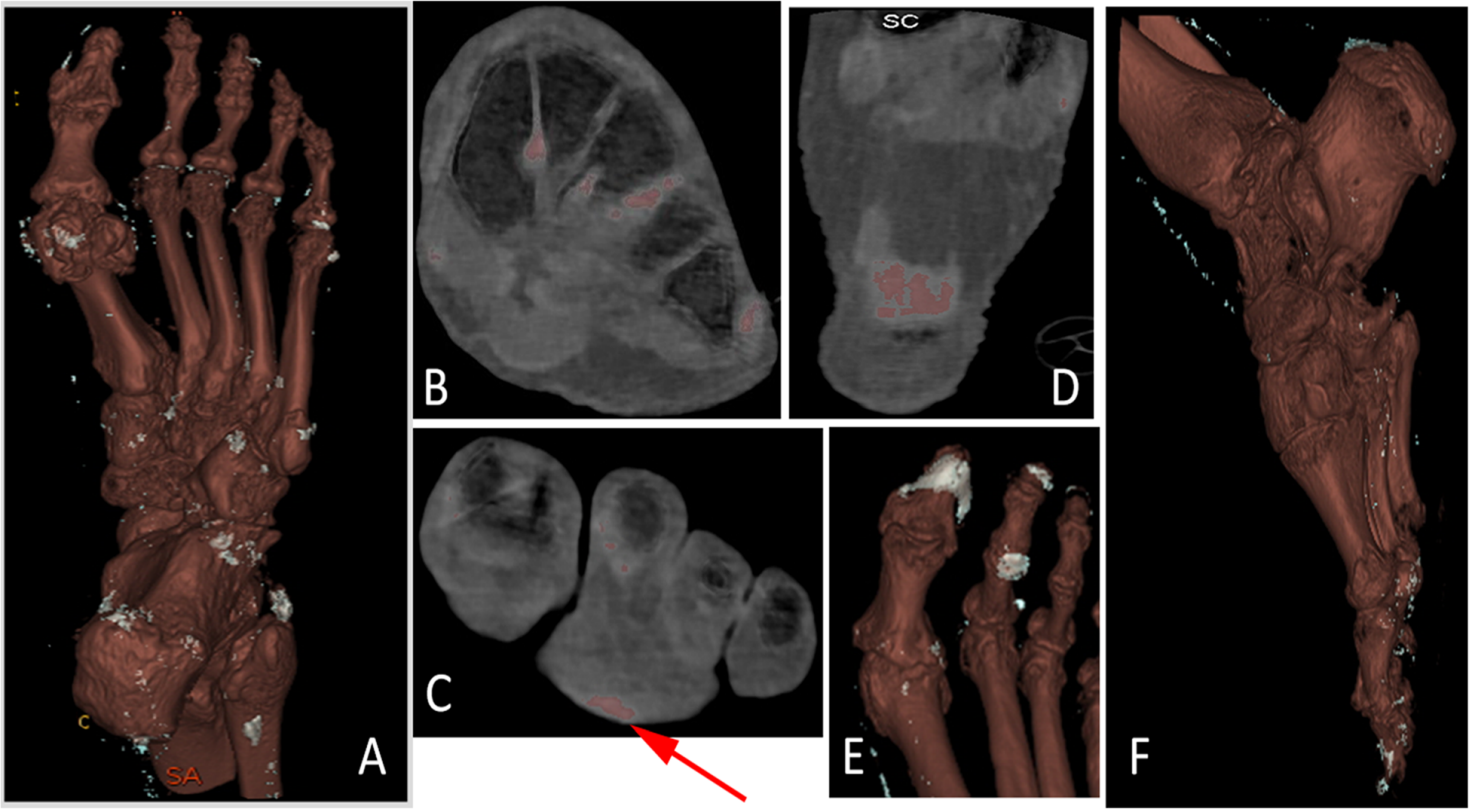
**a**-**f** figure depicting typical findings of urate deposition and typical artifacts. 3D reconstruction, scoring of the foot of a patient with a total score of 24 **a** and the axial scan of the same right midfoot **b**. Artifacts from the skin (red arrow) **c** and note the high amount of colour coded urate in the Achilles tendon insertion **d** and nail bed artifact **e**. Example of scoring, score 3 for tendons in the foot **f**

All patients displayed urate deposits on DECT GSI of the feet, most commonly seen in the MTP-joints but also often present in ankle/midfoot joints and tendons (Figs. 1 and 2, Table 2). Women tended to have more deposits, especially in the tendons, although these differences were not significant (Table 2).

**Table 2:** Urate deposits in joints and tendons and presence of artifacts in the total population and stratified by gender

	Total, n = 55	Men, n = 43	Women, n = 12	*p*-value*
All joints and tendons,				
score (SD)	14.0 (5.5)	13.5 (5.4)	16.1 (5.4)	0.09
MTP1,				
n positive (%)	55 (100)	43 (100)	12 (100)	
score (SD)	4.0 (1.5)	3.9 (1.5)	4.2 (1.7)	0.5
MTP 2–5,				
n positive (%)	55 (100)	43 (100)	12 (100)	
score (SD)	3.9 (1.7)	3.8 (1.8)	4.2 (1.7)	0.6
Ankle/midfoot,				
n positive (%)	53 (96)	42 (98)	11 (92)	
score (SD)	3.6 (1.8)	3.5 (1.8)	3.8 (2.0)	0.6
Tendons,				
n positive (%)	41 (75)	31 (72)	10 (83)	
score (SD)	2.5 (2.3)	2.2 (2.2)	3.5 (2.5)	0.1
Nailartifact,				
n positive (%)	40 (73)	32 (74)	8 (67)	
Skinartifact,				
n positive (%)	17 (31)	12 (28)	5 (42)	
Beam hardening,				
n positive (%)	1 (1.8)	1 (2)	0	
Noise, n positive (%)	0	0	0	

*p-value comparing men and women

The majority of patients (73%) displayed nail artifacts while skin artifacts only were seen in 31% (Figs. 1 and 2, Table 2). Beam hardening was only found in one patient and noise was not seen at all (Fig. 1, Table 2).

Agreement between the two combined readings of DECT images were good (Κappa =0.66, 95% CI = 0.61–0.71). The total urate deposit score was significantly higher in the presence of tophus (*p* = 0.0005) and correlated strongly to disease duration (Spearman Rho 0.64, *p* < .0001) while no association or correlation was seen to age, gender, erosive disease, urate levels, BMI, diuretic use, ULT use or renal function (Table 3).

**Table 3:** Associations between total urate deposit score and demographics, gout characteristics, diuretic and ULT use, BMI and renal function

Parameter	n	Total urate deposit score correlation coefficient
Age	55	−0.23
*p*-value		0.09
Disease duration, years	55	0.64
*p*-value		<.0001
Urate at DECT	55	−0.12
*p*-value		0.36
	n	Total urate deposit score, mean
Age categories	55	
< 65 years	24	13.6 (5.8)
> 65 years	31	14.4 (5.3)
*p*-value		0.5
Gender	55	
Men	43	13.5 (5.4)
Women	12	16.1 (5.4)
*p*-value		0.09
Tophus	55	
Yes	7	21.1 (3.2)
No	48	13.0 (5.0)
*p*-value		0.0005
Erosive disease	55	
Yes	36	14.3 (5.6)
No	19	13.6 (5.6)
*p*-value		0.7
Presence of nail artifacts	55	
Yes	40	14.3 (4.0)
No	15	13.5 (4.5)
*p*-value		0.7
Presence of skin artifacts	55	
Yes	17	15.6 (5.3)
No	38	13.3 (5.5)
p-value		0.2
Disease duration, years	55	
Short ≤7	35	11.9 (4.8)
Long > 7	20	17.8 (4.6)
*p*-value		0.0002
Urate at DECT	55	
≤ 405 μmol/L	19	15.1 (5.5)
> 405 μmol/L	36	13.5 (5.5)
*p*-value		0.18
ULT use at DECT	55	
Yes	22	14.9 (5.4)
No	33	13.5 (5.5)
*p*-value		0.2
Diuretic use	55	
Yes	16	13.9 (5.1)
No	39	14.1 (5.7)
*p*-value		0.8
BMI	42	
< 30	22	13.6 (6.1)
≥ 30	20	13.4 (5.4)
*p*-value		0.9
Renal function by eGFR	55	
≥ 90	16	13.8 (5.2)
60–90	20	12.8 (5.3)
< 60	19	15.5 (5.8)
*p*-value		0.4

## Discussion

In the present study we show that DECT GSI identified urate deposits in the feet of all 55 participants with gout, most commonly seen in the MTP-joints but also often in ankle/midfoot joints and tendons. Furthermore, more depositions were identified in patients with one or more tophi and longer disease duration of gout. Artifacts were mostly found in the nails, a minority displayed skin artifacts while beam hardening and noise was almost not seen at all. The agreement between the two repeated consensus readings was good.

The DECT GSI method has been methodically tested and shown to be able to detect MSU deposits by Li et al. 2014 [12]. Kiefer et al. compared single source DECT with conventional CT and digital radiography and showed that it had the best diagnostic properties of the three for gout diagnosis [13]. However, the vast majority of DECT studies on urate deposition have been performed with dual source machines. For example, Dalbeth et al. [14] showed that the presence of tophi and disease duration were associated with both presence and amount of MSU deposits seen with dual source DECT. Furthermore, they also demonstrated that higher urate levels, lower allopurinol dose, higher number of attacks and attack within three months were associated with higher amounts of MSU deposits. In our study we found no correlation to use of allopurinol but that may be explained by suboptimal dosing of ULT in the present setting, as we have demonstrated before [15].

Artifacts are common in DECT for MSU deposition identification but they are usually readily recognizable. In our study we found quite low numbers of skin artifacts and noise compared to dual source examination [16]. Furthermore, artifacts in the form of beam hardening was only seen in one individual in the present study and motion artifacts were not seen at all. The latter is probably explained by the rapid reconstruction algorithms made possible by the ultrafast scintillator detector of the DECT GSI equipment [16, 17].

There are several strengths to the present study. First, all images were evaluated by two DECT radiologists in two repeated combined readings. Second, the agreement between readings was good. Third, we correlated the DECT findings with extensive data retrieved from medical records.

There are also limitations to our study. First, the study was cross-sectional and therefore not able to identify changes in deposition over time in relation to ULT. Second, in the medical records we did not have reliable data on gout attack characteristics, such as severity and frequency of attacks. Third, almost all subjects were patients at a rheumatology clinic and thus possibly including patients with more severe gout than what is on average seen in primary care. Fourth, the lack of a control group without gout limited the estimation of artifacts. Fifth, examination of the study subjects with other DECT techniques would have rendered important information but was not possible due to the retrospective design of the study. We are however planning a prospective study for such comparisons. Finally, validity was not tested against microscopy of tissue samples collected at sites where DECT indicated deposition of urate. However, such studies have been performed previously, although only on dual source CT scanners, and in all cases shown both high sensitivity and specificity [3, 18].

DECT is an important tool to help clinicians when diagnosing gout but availability is limited due to costs and lack of radiologists. It is therefore important to gain knowledge on the performance of the different available DECT techniques to eliminate that as an obstacle in implementing the procedure. Thus, there is a need for comparative studies between the different DECT techniques and their ability to detect MSU deposits.

To conclude, in the present study we show that DECT GSI performs well in detecting MSU deposits with a low frequency of artifacts in gout patients and amount of deposits correlate with disease duration and presence of tophus.

## Data Availability

The datasets used and/or images analyzed during the current study are available from the corresponding author on reasonable request.

## Abbreviations

BMI: body mass index
DECT: dual energy CT
GSI: gemstone scintillator detector
HT: hypertension
IHD: ischemic heart disease
MSU: monosodium urate
ULT: urate lowering therapy
